# How to dose follitropin delta for the first insemination cycle according to the ESHRE and ASRM guidelines; a retrospective cohort study

**DOI:** 10.1186/s13048-022-01079-w

**Published:** 2023-01-27

**Authors:** Suha Arab, Russell Frank, Jacob Ruiter, Michael H. Dahan

**Affiliations:** 1 Department of Obstetrics and Gynecology, Division of Reproductive Endocrinology and Infertility Center, McGill University, 888 Boulevard de Maisonneuve East, Suit # 200, Montreal, QC H2l 4S8 Canada; 2 Department of Obstetrics and Gynecology, McGill University, Montreal, QC Canada

**Keywords:** IUI, Follitropin Delta, Rekovelle, COH, Gonadotropin, AMH

## Abstract

**Background:**

Follitropin Delta (FD) is indicated exclusively for in-vitro fertilization however, being a gonadotropin it could be used for other purposes. A dosing algorithm exists for FD and IVF but is needed for intrauterine insemination (IUI) cycles. The objective of this study is to determine dosing for FD for the first controlled ovarian hyperstimulation (COH) cycle according to current stimulation guidelines.

**Results:**

A retrospective study of 157 subjects from a single university fertility center from January 2017 to March 2020, was performed. All patients stimulated with FD for IUI were included. The number of failed, normal, or overstimulation cycles was determined based on stimulating not more than 2 mature follicles. We then stratified the group based on the AFC, AMH, and body weight. Of 157 subjects, 49% stimulated correctly, 5.6% failed and 45.4% overstimulated. An analysis of the COH IUI cycles based on stratification and over or lack of stimulation per published guidelines found that women with a bodyweight < 80 kg or AMH ≥ 1.5 ng/ml or AFC ≥ 10 initially stimulate with FD 2.0 to 3.0mcg daily. For women with an AFC of 6–9 stimulate with Follitropin Delta 3.0mcg daily. For women with an AFC < 6 or serum AMH < 1.5 ng/ml stimulate with FD 3.0–4.0mcg daily. For women with body weight > 80 kg stimulate initially with daily with 4.0–6.0mcg FD.

**Conclusions:**

Follitropin Delta can be used safely for controlled ovarian stimulation and insemination at doses easily dispensed by the current methods of delivery, within the current published guidelines for follicle development.

## Background

Controlled ovarian hyperstimulation (COH) with gonadotropins in combination with intrauterine insemination (IUI) has been used as a treatment for infertile couples for many decades. The goal of this treatment is to induce single follicular recruitment in anovulatory women who have failed oral medications [1] or to recruit one or more than one follicle in ovulatory women, to increase the chance of one fertilizing, which is controversial. The issue with multi-follicular recruitment is the risk of multiple pregnancies [2]. This risk can be limited in in-vitro fertilization cycles by transferring a single embryo. However, in COH and IUI cycles this risk may be elevated. As such, many societies have suggested that the recruitment of a single follicle in most cases of COH IUI is ideal [1]. However, because single folliculogenesis is difficult to obtain, most societies currently permit the recruitment of up to 2 or 3 mature follicles before cycles should be abandoned [1]. This includes both the American Society of Reproductive Medicine (ASRM) guidelines for ovarian stimulation in anovulatory women and to minimize risks of multiple pregnancies [3] and The European Society of Human Reproductive (ESHRE) guidelines on decreasing the risk of multiple pregnancies[1].

The use of gonadotropins for COH IUI has become controversial due to the increased risks of multiple pregnancies when compared to oral medication particularly aromatase inhibitors and clomiphene citrate [1]. However, gonadotropin insemination continues to be used. Particularly in women with very thin uterine linings on oral medications or for those patients who failed to respond to oral medications with ovulation. [4, 5]. In a large study of 50,473 stimulated IUI cycles performed in women less than 40 years of age, a cut-off of two mature follicles was found to limit the risk of twin pregnancy, obtained good conception outcomes, and minimized the risk of higher-order multiple pregnancies [6].

Follitropin Delta (FD) (Rekovelle, Ferring Pharmaceuticals, Montreal, Canada) is a novel human cell-derived gonadotropin that has been used to stimulate ovarian follicular growth in-vitro fertilization (IVF) cycles exclusively [7, 8]. FD demonstrated different pharmacokinetics and pharmacodynamics than the other recombinant medication on the market [9]. FD was found to have higher stimulation per international unit and lower serum clearance than follitropin alfa [9]. This is likely due to the assay to calculate international units being a rodent-based assay and the medication functioning in humans and being produced in a human cell line. Follitropin alfa and delta have the same amino acid FSH sequences, however, they have different glycosylation patterns [9]. FD is synthesized in a human cell line while the other recombinant gonadotropins available on the market are synthesized in rodent cell lines, resulting in different protein folding patterns due to the cells of origin [9]. FD has a higher ratio of tri- and tetra-sialylated glycans with both alpha 2,3- and alpha 2,6-linked sialic acid [9]. FD has a more acidic isoform and a lower isoelectric point than other recombinant FSH [9]. Using FD for IVF is based upon a specific dosing regimen, which takes into consideration body weight and serum AMH levels [10] [11] [12]. Being that gonadotropins in general are commonly used for COH IUI, several physicians in the university center decided to use FD for COH IUI. No literature currently exists concerning a dosing algorithm for controlled ovarian hyperstimulation using FD for COH in IUI cycles. Therefore, the objective of our study is to determine a starting dose for FD in women performing COH IUI to obtain stimulation of follicles consistent with the ASRM and ESHRE guidelines.

## Material and methods

This is a retrospective cohort study performed at a single academic infertility center from January 2017 to March 2020 inclusive. McGill University Health Center research ethics board (MUHC REB) approval to carry out and data collection for this study was obtained (2021–6713).

We performed this study to develop a dosing algorithm for FD according to current ASRM and ESHRE guidelines for COH IUI. For this study, we considered overstimulation when ≥ 3 follicles ≥ 10 mm or > 2 follicles ≥ 16 mm in mean diameter are present at the time of triggering. The most recent ESHRE recommendation for COH with or without IUI suggests canceling when > 3 mature follicles are present (size unstipulated), however, it was published in 2000 and may be dated. The ASRM guidelines for COH in anovulatory women as well as the ASRM guidelines to minimize risks of multiple pregnancies suggest cancellation when > 2 mature follicles at least 16 mm in diameter or more than 2 follicles of at least 10 mm are stimulated or more than 3 follicles at least 14 mm in diameter, respectively.

A total of 157 subjects were studied. Physicians prescribed FD at any dose they saw fit based on body weight or ovarian reserve parameters and clinical experience. In general women with normal ovarian reserve and body weight were stimulated with 2 to 4 mcg daily. While women that had excessive body weights or low ovarian reserve were stimulated with 3 to 12 mcg daily depending on the physician prescribing the treatment. At least 4 different physicians treat subjects in our clinic with FD for IUI cycles.

Inclusion criteria: All patients aged 21–42 years who were stimulated with FD for an IUI cycle with at least one patent fallopian tube, no intra-cavity pathology, and ≥ 5 million total motile sperm count. Subjects were included only for their first FD, and IUI cycle. This could be in any of their first three IUI cycles if other gonadotropins had previously been used, not including FD.

Exclusion criteria: Patients with 3 or more failed IUI cycles were not included (all previous cycles had been performed with a different gonadotropin than FD). Subjects with no patent fallopian tubes, stage 3 or 4 endometriosis, or untreated intrauterine pathology, were not in the analysis. All patients had a duration of infertility < 3 years.

All subjects underwent a full infertility evaluation before the start of the stimulation which included baseline hormonal profiles, ovarian reserve testing including either serum AMH levels or antral follicle counts (often both, but not always), transvaginal ultrasound evaluations for uterine and ovarian pathology, demonstration of fallopian tube patency, and semen analysis.

Please note that not all subjects had serum AMH levels or body weight recorded in their files, these were a subset of subjects and were evaluated and recorded as such.

### AFC measurement and AMH assay

All patients had attended the clinic for pelvic examination and baseline transvaginal ultrasound within 6 months of starting the ovulation stimulation treatment for their IUI cycles. Transvaginal ultrasound was performed using a 5 MHz vaginal probe (Voluson 730, GE Healthcare, Wisconsin, USA) to determine the antral follicle count size (2–9 mm in diameter) in both ovaries combined on Day 2 to Day 5 of their spontaneous or progesterone provoked menstrual cycles. This assessment was done at the ultrasound department at the MUHC-glen hospital by one of two certified sonographers, who had > 10 years of experience with pelvic transvaginal ultrasonography.

The AMH analysis was performed using the Elecsys ® assay (Roche Diagnostic GmbH, Mannheim, Germany). This is an immunoassay for the in-vitro quantitative determination of anti-Mullerian hormone (AMH) in human serum and plasma. The electrochemiluminescence immunoassay (ECLIA) is intended for use on the cobase 801 immunoassay analyzer. The Elecsys ®AMH assay has a sensitivity of 0.01 ng/ml (0.0714 pmol/L), and intra-assay and inter-assay coefficients of variation of less than 5.4%. The AMH evaluation was done within 6-months of starting the IUI stimulation.

### Protocol for controlled ovarian stimulation

Follicular stimulation with FD started on the third day of the menstrual cycle after performing a baseline transvaginal ultrasound to rule out an ovarian cyst. If a functional or new ovarian cyst or a follicle greater than 10 mm in diameter was present at that point, the medication was withheld until an evaluation in the subsequent menstrual period. The FD dose was not adjusted in any of the subjects, irrelevant to the patient's response as noted with transvaginal ultrasound monitoring. When the dominant follicle mean diameter reached 18 mm, a recombinant human chorionic gonadotropin trigger was administrated (250 mcg SQ, Merck Serono, Montreal, Canada), and 24 or 36 h later IUI was performed.

Luteal support was prescribed to all patients with 200 mg micronized progesterone daily (Prometrium, Schering Inc., Pointe-Claire, Canada), administration vaginally from the second day after insemination until pregnancy test and beyond if pregnant (until 10 weeks of pregnancy).

According to protocol and based on mean perpendicular measurements by transvaginal ultrasound (Voluson 730, GE Healthcare, Wisconsin, USA) we defined the cycle stimulation outcomes as:

- 1) Overstimulation: when there were > 2 follicles sized 10–18 mm on the day of the hCG trigger, with at least one follicle of 18 mm.
- 2) Failed stimulation: when there was no dominant follicle that grew after FD daily injections for 15–21 days.
- 3) Adequately Stimulated: when 1- 2 follicles with a mean diameter ≥ 10 mm and at least one follicle 18 mm in mean diameter as measured in two perpendicular planes on the day of hCG triggering were present.

We stratified the subjects group based on the ovarian reserve parameters (AFC, AMH), and body weight to determine if an initial starting dose could be determined for the insemination cycles using FD.

### Statistical analysis

All data were presented as mean ± SD or percentage. Data was stratified using descriptive statistics. All calculations were performed using SPSS version 23.0 (IBM Corporation, Chicago, USA). A comparison of overstimulation rates was performed using chi-squared tests.

## Results

The patients' mean age was 37.0 ± 4.2 years, the partners' mean age was 39.0 ± 5.9 years, the mean total motile sperm count pre-insemination was 37.0 ± 44.6 million, basal serum FSH 9.9 ± 9.1 IU/L, serum TSH 1.7 ± 0.8 IU/L, serum AMH 2.5 ± 2.8 ng/ml, AFC 13.1 ± 10.7 and patient weight 75.9 ± 21.0 kg. The causes of infertility were listed in Table 1.

**Table 1:** Causes of infertility among all subjects:

Decrease ovarian reserve DOR	40%
Endometriosis (stage 1 or 2)	9%
Male factor	29%
PCOS	9%
Single (no partner)	2%
Unexplained	9%
Other	2%

Among all subjects 49% were stimulated per protocol, 5.6% failed to stimulate, and 45.4% were overstimulated. For the sake of the following data, FD doses are per day.

The results of the stratified outcomes per dose group for ovarian stimulation are presented in Table 2.

**Table 2:** Ovarian stimulation response among all subjects in general and stratified

	**Correctly Stimulated**	**Over-Stimulated**	**Failed to Stimulate**
Overall 157 subjects	49%	45.4%	5.6%
**Serum AMH Stratified (*N* = 84)**			
**AMH ≥ 1.5 ng/ml**			
FD dose 2- 3 mcg (*N* = 29)	79%	17.6%	3.4%
FD doses > 3 -12mcg (*N* = 29)	20%	62%	18%
**AMH < 1 ng/ml**			
FD dose ≤ 4 mcg (*N* = 5)	75%	25%	0%
FD doses 6–12 mcg (*N* = 31)	29%	48%	23%
FD dose 4- < 6 mcg (*N* = 0)	NA	NA	NA
**AMH ≥ 1 and < 1.5 ng /ml**			
FD dose 3–12 mcg (*N* = 19)	53%	47%	0%
FD dose 3-6mcg (*N* = 9)	67%	33%	0%
FD dose 3–4 mcg (*N* = 6)	80%	20%	0%
FD dose 7–12 mcg (*N* = 10)	40%	60%	0%
FD dose 6–7 mcg (N = 0)	NA	NA	NA
**AFC Stratified (*N* = 134)**			
**AFC ≥ 10**			
FD dose 2–12 mcg (*N* = 78)	52%	40%	8%
FD dose > 3 mcg (*N* = 37)	29%	60%	11%
FD dose 2- 3 mcg (*N* = 41)	73%	24.6%	2.4%
**AFC < 6**			
FD dose 3–12 mcg (*N* = 36)	44%	50%	6%
FD dose 3- 4 mcg (*N* = 6)	50%	50%	0%
FD dose > 4–12 (*N* = 30)	44%	47%	9%
FD dose 3 mcg (*N* = 3)	100%	0%	0%
**AFC 6–9**			
FD dose 3–12 mcg (*N* = 12)	25%	75%	0%
FD dose 6–12 mcg (*N* = 17)	12%	88%	0%
FD dose 3mcg (*N* = 3)	100%	0%	0%
**Body Weight Stratified (*N* = 71)**			
**Weight** ≥ **80 kg**			
FD dose 3–12 mcg (*N* = 26)	50%	27%	23%
FD dose 4–6 mcg (*N* = 20)	50%	25%	25%
FD dose > 6 mcg (*N* = 6)	50%	33%	17%
**Weight < 80 kg**			
FD dose 2–12 mcg (*N* = 45)	47%	53%	0%
FD dose 2–4 mcg (*N* = 17)	71%	29%	0%
FD dose 5.33–12 mcg (*N* = 28)	29%	71%	0%
FD dose 4–5.33 (*N* = 0)	NA	NA	NA

### Ovarian stimulation response stratified for AFC

AFC was available on 134 subjects. Among subjects with AFC ≥ 10 (*N* = 78), doses ranged from (2–12 mcg daily), 53% stimulated per protocol, 8% failed to stimulate, and 40% overstimulated. Among AFC ≥ 10 & FD dose ≤ 3mcg daily (range 2–3 mcg), (*N* = 41): 73% stimulated per protocol, 2.4% failed to stimulate and 24.6% overstimulated. Among women with AFC ≥ 10 & FD dose > 3 mcg daily (range 4.00 to 12 mcg) (*N* = 37): 29% stimulated per protocol, 11% failed to stimulate, and 60% overstimulated. This comparison did not take into consideration body weight or serum AMH.

For women with an AFC 6–9 (*N* = 20), 25% were stimulated per the protocol, and 75% were over-stimulated, dose range 3–12 mcg daily. For those with AFC 6–9 and dose 6-12mcg daily (*N* = 17), 12% were stimulated per the protocol and 88% were over-stimulated, 0% failed to stimulate. For those with AFC 6–9 and stimulated with 3mcg daily (*N* = 3) 100% stimulated per protocol, 0 were over-stimulated and 0 failed to stimulate. None of these subjects were stimulated with between 3.3 and 5.6 mcg daily. Weight was not taken into consideration for this analysis, nor was serum AMH.

Among women with AFC < 6 (*N* = 36); daily doses ranged (3-12mcg), 44% stimulated per protocol, 50% over-stimulated, and 6% failed to stimulate. Among women with AFC < 6 and FSH dose ≤ 4mcg (3–4 mcg, range) (*N* = 6): 0% failed to stimulate, 50% stimulated per the protocol, and 50% over-stimulated. Among women with AFC < 6 and FD dose 4.3 to 12 mcg (*N* = 30); 47% were over-stimulated and 47% were stimulated per the protocol and 7% failed to stimulate. Among women with AFC < 6 and FSH dose = 3 mcg (*N* = 3): 100% stimulated per protocol. This analysis did not consider body weight or serum AMH.

### Ovarian stimulation response stratified for AMH

AMH levels were available on 113 subjects. Among women with serum AMH ≥ 1.5 ng/ml and FD dose of 2 or 3mcg daily (*N* = 29), 79% stimulated per protocol, 17% over-stimulated, and 3.4% failed to stimulate. Among women with serum AMH ≥ 1.5 ng/ml and FD dose > 3 mcg daily (range 3.66–12.0 mcg) (*N* = 29) 62% were over-stimulated, 20% were stimulated per the protocol and 18% failed to stimulate.

Among women with serum AMH ≥ 1.0 ng/ml and < 1.5 ng/dl (*N* = 19) and FD dose range of 3 to 12 mcg, 53% were stimulated per the protocol and 47% over stimulated. Among women in this group stimulated with 3 to 6 mcg daily (*N* = 9). 67% stimulated per the protocol and 33% over-stimulated, 0 failed to stimulate. Among women in this group stimulated with 3 to 4 mcg daily 20% were over-stimulated and 80% stimulated per protocol (*N* = 6). Among women stimulated with 7–12 mcg daily (*N* = 10) 60% over stimulated and 40% were stimulated per protocol. None of the patients with AMH ≥ 1.0 ng/ml and < 1.5 ng/dl failed to stimulate. None of the subjects were stimulated with a FD dose between 6 and 7 mcg daily. This comparison did not consider body weight.

Among women with serum AMH < 1.0 ng/ml and FD dose ≤ 4mcg (range 3.0–4.0 mcg) (*N* = 5) 75% were stimulated per the protocol and 25% were over-stimulated, 0% failed to stimulate. Among women with AMH < 1.0 ng/ml and FD dose 6.0 to 12.0 mcg daily (*N* = 31): 29% were stimulated per protocol, 48% were over-stimulated, and 23% failed to stimulate. None of the subjects received a FD daily dose between 4.3 and 5.6 mcg daily.

### Ovarian stimulation response stratified for body weight

Bodyweight measurements were available on 71 subjects. Among women with body weight ≥ 80 kg (*n* = 26, range 81.0–129.7 kg), dose range (3-12mcg daily) 50% stimulated per protocol, 23% failed to stimulate, and 27% over-stimulated.

Among women with body weight ≥ 80 kg and FSH dose was 4-6mcg daily (*N* = 20, range 81.6–118.8 kg), 50% were stimulated per protocol, 25% failed to stimulate, and 25% over-stimulated.

Only 6 subjects were stimulated with doses greater than 6 mcg daily with bodyweight ≥ 80 kg (range 81.0–129.7 kg), 33% over-stimulated and 50% stimulated per the protocol, and (*N* = 1) failed to stimulate.

Among women with body weight < 80 kg (*N* = 45, range 47.6–77.1), 47% were stimulated per the protocol, and 53% over stimulated with a dose range of FD 2–12 mcg daily. All these patients were stimulated.

Among women with body weight < 80 kg and FD dose 2 to 4 mcg daily (*N* = 17, range 47.6–77.1), 71% were stimulated per the protocol, and 29% were over-stimulated with a dose range of FD 2–12 mcg daily. All of the patients stimulated follicular growth.

Among women with body weight < 80 kg and FD dose 5.33 to 12.0 mcg daily (no subjects were treated with a dose between 4 and 5.33 mcg daily of FD) (*N* = 28, range 55.0–76.0 kg) 71% over-stimulated and 29% stimulated per protocol.

Stimulation responses are summarized in Table 2

Based on the data above the following recommendations for COH IUI using FD doses were developed for the first cycles.

- 1) For women with a bodyweight < 80 kg stimulate initially with daily with 2.0–4.0 mcg FD
- 2) For women with body weight > 80 kg stimulate initially with daily with 4.0–6.0 mcg FD
- 3) For women with an AFC ≥ 10 stimulate with 2.0–3.0 mcg daily
- 4) For women with AFC of 6–9 stimulate with 3.0 mcg daily
- 5) For women with an AFC < 6 stimulate initially with 3.0–4.0 mcg daily
- 6) For women with serum AMH < 1.5 ng/ml stimulate with FD 3.0–4.0 mcg daily

- 7) For women with serum AMH ≥ 1.5 ng/ml stimulate with FD 2.0–3.0 mcg daily
  - If different parameters conflict, we recommended using the higher range of FD dose based on the different parameters listed above.
  - For the first cycle we recommended initiating stimulation with the lowest dose from the range.
  - Titrate FD dose based on response. If no stimulation occurs within 5 days; confirmed by a failure to increase serum estradiol level or develop follicles at least 10 mm in mean diameter, gonadotropin doses can be increased in the range or above the range if the higher doses in the range listed above have failed to stimulate folliculogenesis.

A combined representation of our dose recommendations is included in Table 3.

**Table 3:** The daily dosing algorithm for the first Follitropin Delta controlled ovarian stimulation cycle for intercourse or insemination

AFC	< 6	6–9	≥ 10
Weight ≥ 80 kg	6 mcg	4–6 mcg	4 mcg
Weight < 80 kg	3–4 mcg	3 mcg	2-3mcg
AMH	**< 1.5 ng/ml**		**≥ 1.5 ng/ml**

We compared women who were stimulated based on these dose recommendations (Table 3) for Antral follicle Counts. Those stimulated with the recommended doses of FD were compared to those stimulated with higher doses for rates of stimulation per protocol vs. over-stimulation. Rates of overstimulation were statistically higher *p* = 0.0004 if these dose guidelines were not followed.

We also compared outcomes based on AMH and those stimulated based on recommended doses were statistically less likely to overstimulate and more likely to stimulate per guidelines as compared to those stimulated with higher doses (*p* < 0.0001).

When we compared dosing based on body weight. We found that those who were stimulated per our recommendations provided in Table 3 were less likely to over-stimulate and more likely to stimulate per guidelines as compared to women stimulated with higher doses of FD (*p* = 0.004).

The pregnancy rate for all comers irrespective of age, ovarian reserve, and diagnosis was 11% per insemination performed. Among the subjects that were stimulated with our recommended doses, multiple pregnancy rates were 7.9%. The data of multiple pregnancy rates in women with over-stimulation is biased by conversions to IVF and cancelations with few completing the inseminations, limiting the possibility for results.

## Discussion

Due to its cell line of production and protein folding, FD has a different affinity for the human FSH receptor than other recombinant gonadotropins on the market [13]. This difference in affinity and the fact that the assay is calculated in a rodent cell line has led to the Steelman and Pohley assay under-representing the activity of FD in humans [3]. To prevent confusion on the part of clinicians leading to excessive ovarian stimulation, FD is dosed based on mcgs. However, there are no dosing recommendations for FD for COH and IUI cycles. The literature would suggest that 10 mcg of FD is equivalent to about 150 IU of r-FSH [14]. This would suggest that for the IUI cycle women should be treated with about 1/3 to half this dose or 2.5 to 5 mcg daily for COH IUI. However, our data suggest that this dose would over-stimulate many subjects.

We used a combination of the ASRM recommendations for COH in anovulatory women and the ESHRE guidelines for minimizing risks of multiple gestations when using COH to determine what cutoff to use as over-stimulation in our COH subjects. It should be noted that the number of follicles we selected was consistent with the ASRM guidelines and slightly conservative but consistent with the ESHRE guidelines. However, the ASRM guidelines are relatively recent, while the most up-to-date ESHRE guidelines date from 2000, which is why we took the penchant described. The selected cutoff is also consistent with the limit for follicles recommended in the largest study to date of multiple pregnancies and COH IUI [11]. If the treating clinician wants to develop a greater number of follicles, slightly higher doses of FD should be used than recommended by us. However, our recommendations still act as a guideline on which the doses can be increased by 0.6 to 1.0 mcg above our recommendations. However, we would caution that there could be high multiple pregnancy rates in those cases. Based on the dosing regiments we suggested, in some cases around 25% would over-stimulate. However, it should be noted that we do not know the rate of overstimulation based on the ASRM or the ESHRE recommendations for women undergoing their first COH IUI cycle using gonadotropins. It may be equivalent to or even higher than what occurred in our study in real-life scenarios. Data from the large, cited study of 24,649 women performing 50,473 suggests that about 34% over stimulate [6].

Pregnancy data and multiple pregnancy data are beyond the scope of this article for several reasons. One is that the study is underpowered to address these issues. The second issue is that doses of FD used for COH in our study were often much higher than our final recommendations, artificially increasing the risk of multiple pregnancies. However, among the subjects that were stimulated with our recommended doses, multiple pregnancy rates were under 8% in our cohort. The pregnancy rate for all comers irrespective of age, ovarian reserve, and diagnosis was 11% per insemination cycle performed.

It will be fascinating to see how our proposed starting doses function in women with Polycystic Ovary Syndrome (PCOS). A previous study using a nomogram for gonadotropin dose for COH for IUI found that women with PCOS were often under-stimulated [15]. Although this finding was based on 9 women. Given the increased production of AMH per follicle in women with PCOS, it was felt that too-low doses were selected in this cited study [15]. It should also be acknowledged that insulin-sensitizing agents in women with PCOS and the type of said agent can alter ovarian stimulation in both a positive and a negative manner [16]. Such agents were not used by any of the patients in this study, possibly altering dosing recommendations if used.

In our study obese patients (BMI ≥ 80 kg/m^2^) required higher doses of FD. This is not surprising since the literature supports the use of up to twice as high doses for ovarian stimulation being required in Obese as compared to lean subjects [17].

Endometriosis is another pathology that may alter COH IUI pregnancy outcomes. Current recommendations include COH with or without IUI, particularly in stage 1 or 2 endometriosis [18]. Although, some studies have treated women with failure to conceive with surgically treated endometriosis directly by IVF bypassing the COH [19].

The strength of the study includes a moderate sample size, that this is the first study to investigate a dosing regimen using FD in COH IUI cycles, and that stratification for dosing was also performed based on AFC, because AMH levels are not performed in many centers around the world, particularly in Africa and Asia due to costs.

Weaknesses include the retrospective nature, which may mask undetected bias, and the relatively small number of subjects in certain groups used to make dosing decisions. Our findings from our retrospective data should be subsequently validated in a large randomized controlled trial. Our current manuscript presents the follicular development data in the form of stimulated, over-stimulated, and failed to stimulate. Although, some may have issues with the number of follicles we accepted to classify them as over-stimulated. Nevertheless, multiple pregnancy rates in this group were acceptable at 8%. Data on pregnancy, multiple pregnancies, ovarian hyperstimulation syndromes, or other relevant outcomes parameters were not elaborated on for multiple reasons, first pregnancy outcome was not primarily one of the objectives of this study. We wanted first to try to find a dosing protocol for using FD in IUI cycles by evaluating the follicular response. Many cycles were canceled for failure to stimulate or for over-response or converted to IVF, which would skew our results. In a subsequent study with a larger population, we would evaluate the pregnancy, multiple pregnancies, and even possibly the OHSS outcomes in IUI cycles based on our dose recommendations. (No cases of mild, moderate, or severe OHSS occurred in our cohort). It should be noted that we always selected the most conservative recommendations for the initial starting doses. This was done to minimize the risks of multiple pregnancies since doses can always be increased if needed to increase stimulation in a subsequent cycle or to initiate stimulation in the current cycle. Some may feel that we needed institutional review board (IRB) approval to use FD for COH IUI, however, it is common to use medications off-label in healthcare, and as such, we would argue against a prospective IRB being required.

Some may state that our dosing recommendations were conservative. However, in cycles with three or four follicles, the multiple pregnancy rate likely increases substantially without a significant gain in the overall pregnancy rate [6]. Most fertility centers performing COH IUI claim to aim for not more than three follicles or two mature follicles before trigger due to the risk of multiple pregnancies [20]. As such our recommendations for overstimulation are similar to what most fertility centers claim to do. Lastly, not all patients had AFC, AMH levels, or body weight recorded which may theoretically contribute to heterogeneity in results. However, given the significant overlap in recommended FD based on poor prognosis groups including high body weight, serum AMH < 1.5 ng/ml, and AFC < 6, it is unlikely that this heterogeneity affected our results.

One may ask why a body weight of 80 kg was chosen as the cutoff point. When we modeled with lower body weights (such as 70 kg), consistent stimulation patterns failed to appear, suggesting that a body weight of 80 kg was the correct cut-off to select for the dosing recommendations.

## In Conclusion

We present the first recommendations in the medical literature for COH IUI or intercourse using FD.

## Data Availability

Not applicable.

## Abbreviations

AFC: Antral follicle count
COH: Controlled ovarian hyperstimulation
DOR: Diminish ovarian reserve
FSH: Follicle-stimulating hormone
FD: Follitropin delta
HCG: Human chorionic gonadotropin
IUI: Intrauterine insomination
IVF: In vitro fertilization
PCOS: Polycystic ovarian syndrome
